# Mechanism and Molecular Targets of Ejiao Siwu Decoction for Treating Primary Immune Thrombocytopenia Based on High-Performance Liquid Chromatograph, Network Pharmacology, Molecular Docking and Cytokines Validation

**DOI:** 10.3389/fmed.2022.891230

**Published:** 2022-07-13

**Authors:** Ming Jing Wang, Yan Sun, Ying Song, Ju Ning Ma, Zi Qing Wang, Xiao Qing Ding, Hai Yan Chen, Xue Bin Zhang, Min Min Song, Xiao Mei Hu

**Affiliations:** ^1^Xiyuan Hospital, China Academy of Chinese Medical Sciences, Beijing, China; ^2^Nankou Hospital, Beijing, China; ^3^Dongfang Hospital, Beijing University of Chinese Medicine, Beijing, China

**Keywords:** Ejiao Siwu decoction, primary immune thrombocytopenia, HPLC, network pharmacology, therapeutic mechanism, molecular docking

## Abstract

We explored the mechanisms and molecular targets of Ejiao Siwu Decoction (EJSW) for treating primary immune thrombocytopenia (ITP) using network pharmacology and molecular docking. Active compounds of EJSW were identified by high-performance liquid chromatography-diode array detector (HPLC-DAD) and high-performance liquid chromatography-mass spectrometry (HPLC-MS) and their targets were obtained from HERB and SwissTargetPrediction, and ITP targets were obtained from Comparative Toxicogenomics Database (CTD) and GeneCards. STRING and Cytoscape were used for protein-protein interaction (PPI) network analysis. Gene Ontology (GO) and Kyoto Encyclopedia of Genes and Genomes (KEGG) analyses by WebGestalt yielded a gene-pathway network, Autodock molecular docking was applied to screen targets and active compounds, and cytokines were detected using a cytometric bead array (CBA) human inflammation kit. We identified 14 compounds and 129 targets, and 1,726 ITP targets. RAC-alpha serine/threonine-protein kinase (AKT1), tumour necrosis factor (TNF), interleukin-6 (IL6), caspase-3 (CASP3) and tumour suppressor protein (TP53) were core targets (nodes and edges). Functional annotation identified cofactor binding and coenzyme binding, and 20 significantly enriched pathways. Active compounds of EJSW were successfully docked with ITP targets. Tumour necrosis factor alpha (TNF-α) and interleukin-1 beta (IL-1β) were upregulated in ITP patients, vascular endothelial growth factor A (VEGF-A) and vascular endothelial growth factor D (VEGF-D) were downregulated, and EJSW treatment reversed these trends. EJSW may regulate key ITP targets based on the *in silico* analyses, and protect vascular integrity through AGE-RAGE signalling, complement and coagulation cascades, and VEGF signalling by downregulating TNF-α, IL-1β and other inflammatory factors.

## Highlights

- Active compounds of Ejiao Siwu (EJSW) Decoction were detected by HPLC-DAD and HPLC-MS.
- EJSW may regulate key ITP targets, and protect vascular integrity through AGE-RAGE signalling, complement and coagulation cascades, and VEGF signalling by downregulating TNF-α, IL-1β and other inflammatory factors.
- EJSW treatment reversed the expression of cytokines in patients with ITP.

## Introduction

Primary immune thrombocytopenia (ITP) is an autoimmune disease characterised by platelet destruction, insufficient platelet production, high risk of bleeding, fatigue and immune dysfunction (1–5). Decreased platelet count in ITP patients is caused by pathologic antiplatelet antibodies (6), damaged megakaryocytopoiesis (7), and immune disorders such as altered T helper 1/T helper 2 balance (8), unbalanced Th17/Treg (9), and lower Breg impression (10). Glucocorticoids are still the standard first-line treatment, but they cause serious side effects following long applications (11), and second-line therapies for ITP are still lack of enough reliable data in term of long-term safety and efficacy (12).

Traditional Chinese medicine (TCM) is considered an effective auxiliary strategy for treating chronic diseases, including ITP. It can increase PLT counts and reduce bleeding with significant efficacy (13, 14) and also increase the number of granulocytes megakaryocytes (15). Ejiao Siwu (EJSW) decoction is composed of five Chinese medicinal herbs: Angelicae Sinensis Radix (ASR, the dried root of Angelica sinensis [Oliv.] Diels), Chuanxiong Rhizoma (CR, the dried rhizome of Ligusticum chuanxiong Hort.), Paeoniae Radix Alba (PRA, the dried root of Paeonia lactiflora Pall.), Rehmanniae Radix Praeparata (RRP, the dried root of Rehmannia glutinosa Libosch.), and Asini Corii Colla (ACC, a product from the hide of Equus asinus L.). It has been reported that Angelicae Sinensis Radix, Chuanxiong Rhizoma, Paeoniae Radix Alba, Rehmanniae Radix Praeparata could substantially promote the proliferation of bone marrow haematopoietic cells in anaemic mice (16). Asini Corii Colla possesses a therapeutic effect in treating various haematologic diseases, including thrombocytopenia, which increases PLT counts and stimulates the activity of bone marrow stem cells, especially megakaryocytes (17). EJSW is frequently used to treat blood deficiencies, coughing, and associated symptoms including anaemia, asthenia, dizziness, fatigue, irregular menstruation, palpitations, muscle fatigue and pale complexion. In addition, EJSW can nourish platelets, improve endothelial function, and treat ITP in TCM clinical practice. However, the mechanisms and molecular targets of EJSW for treating of ITP are not yet clear, which is the main factor limiting the application and development of EJSW worldwide.

The chemical components of EJSW were analysed by HPLC-DAD and HPLC-MS techniques. This method is reliable and reproducible, and can more accurately determine the true content of chemical components. It is widely used in laboratories for quality control of traditional Chinese medicines. Network pharmacology is a new discipline integrating systems biology and pharmacology. Using extensive biological information, existing molecular biology data are systematically and comprehensively analysed, and interaction mechanisms linking organisms and diseases are deciphered based on protein abundance, gene expression, and levels of small molecules (metabolites) (18). It can be used for the development and mechanism research of multi-target drugs (19, 20). Most previous research on the pharmacodynamic basis and mechanisms of TCM has focussed on the regulation of single targets or signalling pathways, and it has proved difficult to reveal synergistic effects between TCM components. However, network pharmacology combines drug target network and bioinformatics network analyses from a multi-target perspective, which could help to reveal complicated TCM mechanisms and assist new drug research and development (21, 22).

In the present study, a network pharmacology approach was used to explore the mechanisms and molecular targets of EJSW for the treatment of ITP. Active compounds of EJSW were identified by HPLC-DAD and LC-MS and their targets were obtained using HERB^1^ and SwissTargetPrediction^2^. ITP-related targets were then derived from Genecards and Comparative Toxicogenomics Database (CTD). The mechanisms through which EJSW treats ITP were analysed by Gene Ontology (GO) and Kyoto Encyclopedia of Genes and Genomes (KEGG) pathway analyses. The findings lay a foundation for the clinical application of EJSW to treat ITP.

## Materials and Methods

### Preparation and Quantitative Analysis of the Chemical Components in Ejiao Siwu by High-Performance Liquid Chromatography-Diode Array Detector and High-Performance Liquid Chromatography-Mass Spectrometry

Chinese herbal medicine pieces of ASR, CR, PRA, RRP, ACC and their precision compound granules were provided by the Chinese Pharmacy of Xiyuan Hospital of China Academy of Chinese Medical Sciences. A 2.5 g of precision compound granules were taken and added 20 ml of 50% methanol for ultrasound for 40 min. After cooling, it was weighted with 50% methanol. The supernatant was taken and injected through 0.22 μm filter membrane for quantitative analysis by HPLC-DAD. The contents of gallic acid, albiflorin, paeoniflorin, senkyunolide I, ligustilide, oxidised paeoniflorin, catechin, caffeic acid, verbascoside, chlorogenic acid, alanine, L-proline, glycine and L-hydroxyproline as the markers were detected.

The chemical analysis was conducted on a high performance liquid chromatograph (HPLC, waters-2695, Waters Corporation, Milford, MA, United States) equipped with DAD detector. The column (Diamonsil C18, 250 × 4.6 mm, 5 μm) was used for chromatographic separation. The mobile phase was acetonitrile (B), 0.1% phosphoric acid aqueous solution (A), with a gradient elution procedure (0–3 min, 95%A; 3–15 min, 95–85%A; 15–40 min, 80%–0 A; 40–45 min, 0 A; 45–46 min, 0–95%A; 46–50 min, 95%A). The data was collected by scanning at full wavelength, and chromatograms with detection wavelengths of 276 and 324 nm were extracted, respectively. The flow rate was 1 ml/min, and the column temperature was 35°C, and the injection volume was 10 μL.

The powder of Ejiao was weighed for 2.69 g, accurately added with 25 ml of pure water, weighed, sonicated for 30 min, cooled, weighed, supplemented with pure water, shaken, filtered, and used for HPLC-MS.

The liquid system is Shimadzu ultra-high performance liquid chromatograph (Shimadzu LC30), and the mass spectrometry system is SCIEX 5,600 + mass spectrometer (United States, AB Sciex Instruments, Model TripleTOF 5,600 + Hybrid Quadrupole-TOF LC/MS/MS Mass Spectrometer). The chromatographic column: ACQUITY UPLC HILIC Column 1.7 μm, 2.1 mm × 100 mm; mobile phase: A is 0.1% formic acid water, B is acetonitrile, and the elution program is shown in the following Table 1. The column temperature is 35°C, the sampler temperature is 4°C, and the injection volume is 10 μl.

**TABLE 1:** The gradient elution program for HPLC-MS.

Time (min)	Flow (ml/min)	A% (0.1%FA)	B% (acetonitrile)
0.0	0.3	95	5
3	0.3	95	50
15	0.3	20	100
40	0.3	0	0
45	0.3	0	0

Mass spectrometry conditions: the mass spectrometry adopts positive ion mode (ESI+), independent data acquisition mode (IDA), the collision voltage was 35 V, the capillary voltage was 5,500 v (positive ion), the ion source temperature was 550°C (positive ion), and the de clustering voltage was 60 V. The curtain gas flow was 25 l/min, the gas flow of Gas 1 and Gas 2 were both 50 l/min, and all gases were nitrogen.

### Obtaining Immune Thrombocytopenia-Related Targets

GeneCards (23)^3^ and CTD (24, 25)^4^ were used to identify targets related to ITP, and results overlapping both databases were selected as ITP-related targets.

### Network Construction

A disease-compound-target network was constructed and visualised using Cytoscape 3.7.1^5^ open source bioinformatics analysis software. In the resulting structured network nodes represent genes, proteins or molecules and edges between nodes represent interactions between these biomolecules. The degree of a node represents the connection between nodes in the network. The larger the number and degree, the more likely the target is to be a key target for compounds (26). Protein-protein interaction (PPI) data were obtained from the STRING database^6^, the protein type was set to Homo sapiens, and the lowest interaction threshold was set to medium confidence >0.4 (27). The other parameters were set by default to generate the PPI network, and the top 20 core targets from PPI networks were visualised with R software (Version 3.5.3^7^).

### Bioinformatic Analysis

Gene Ontology analysis of biological process (BP), cellular component (CC) and molecular function (MF) categories was carried out using the WebGestalt web-based gene set analysis toolkit^8^ (28). These functional categories were enriched within various genes (*p* < 0.05), relationships between genes and GO terms were visualised using a chord picture, and relationships among enriched GO terms were visualised using a DAG picture. WebGestalt assigned the KEGG database for pathway analysis, and pathways with significant changes (*p* < 0.05) were subjected to further analysis. Visualisation of the results of GO and KEGG analyses was constructed by Bioconductor (version 3.8^9^) within R (version 3.5.3). Genes that significantly regulated pathways were selected for gene-pathway network analysis, and a gene-pathway network was constructed to screen key target genes through which EJSW treats ITP.

### Molecular Docking of Active Compounds and Core Targets

The 3D structures of active compounds were obtained through the PubChem database^10^. Crystal structures of core target proteins were downloaded from the PDB database^11^, and operations such as dewatering, removing original ligands, adding hydrogens, and calculating charges were performed using PyMOL (version 2.4.0^12^) and autodocktools (version 1.5.6^13^). Molecular docking was performed using autodock Vina^14^, and PyMOL was employed for visualisation.

### Verification of Network Pharmacology-Based Screening Results

In order to confirm the screening results based on network pharmacology, various cytokines including IL-1β, TNF-α, VEGF-A, and VEGF-D were assessed. Twenty-two ITP patients and 15 healthy volunteers (HV) were recruited from Xiyuan Hospital of China Academy of Chinese Medical Sciences (Beijing, China). All patients were treated with EJSW for 2 months. After this period, 2 mL of peripheral blood from ITP patients and HV group members was collected, centrifuged (1,500 rpm, 10 min), and the resulting serum was to assess IL-1β, TNF-α, VEGF-A and VEGF-D by a cytometric bead array (CBA) human inflammation kit according to the manufacturer’s instructions (BD PharMingen, San Diego, CA, United States) (29). Analysis was performed using the AimPlex Bead-based multiparametric flow cytometry instrument (EPICS-Elite, Beckman-Coulter, United States).

This study was approved by the Clinical Research Ethics Committee of Xiyuan Hospital, China Academy of Chinese Medical Sciences (Ethics approval number: 2015XLA108) and was conducted in compliance with the Declaration of Helsinki. All analyses were performed using SPSS 23.0 (SPSS Inc., Chicago, IL, United States). The threshold of significance was defined as two-sided *p* < 0.05.

## Results

### Identification of the Active Compounds in Ejiao Siwu by High-Performance Liquid Chromatography-Diode Array Detector and High-Performance Liquid Chromatography-Mass Spectrometry

**FIGURE 1:**
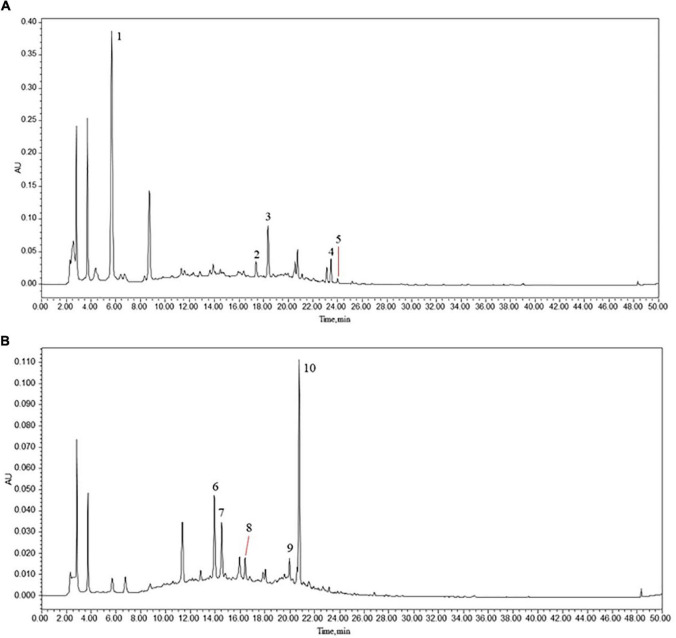
(1) Gallic acid, (2) albiflorin, (3) paeoniflorin, (4) senkyunolide I, (5) ligustilide, (6) oxypaeoniflorin, (7) catechin, (8) caffeic acid, (9) verbascoside, and (10) chlorogenic acid. HPLC-DAD chromatograms of various constituents in EJSW. **(A)** 276 nm. **(B)** 234 nm.

**FIGURE 2:**
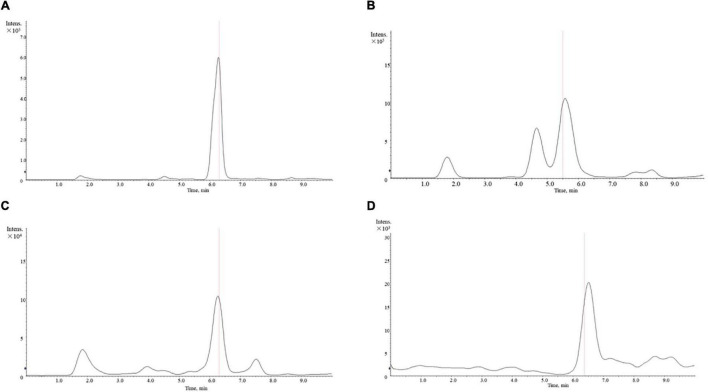
Mass spectrum of amino acid content in ACC. **(A)** Alanine. **(B)** L-proline. **(C)** L-hydroxyproline. **(D)** Glycine.

We obtained a total of 14 active compounds of EJSW and they were gallic acid, albiflorin, paeoniflorin, senkyunolide I, ligustilide, oxidised paeoniflorin, catechin, caffeic acid, verbascoside, chlorogenic acid, alanine, L-proline, glycine and L-hydroxyproline. Among them, 10 active compounds including gallic acid, albiflorin, paeoniflorin, senkyunolide I, ligustilide, oxypaeoniflorin, catechin, caffeic acid, verbascoside, and chlorogenic acid were detected by HPLC-DAD, as shown in Figure 1. A shows the chromatogram of EJSW detected at 276 nm, and B shows the chromatogram of EJSW at 234 nm. The content of other four active compounds (alanine, L-proline, glycine and L-hydroxyproline) in ACC were 28.611, 11.575, 4.749, and 1.989 μg/g, as shown in Figure 2.

### Compound-Target Network Analysis

**FIGURE 3:**
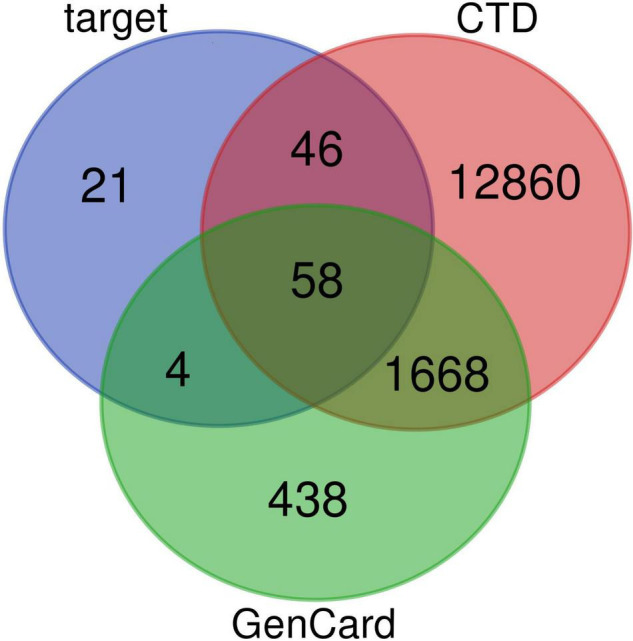
Venn diagram of targets of EJSW and ITP (from Gene Cards database and CTD database).

**FIGURE 4:**
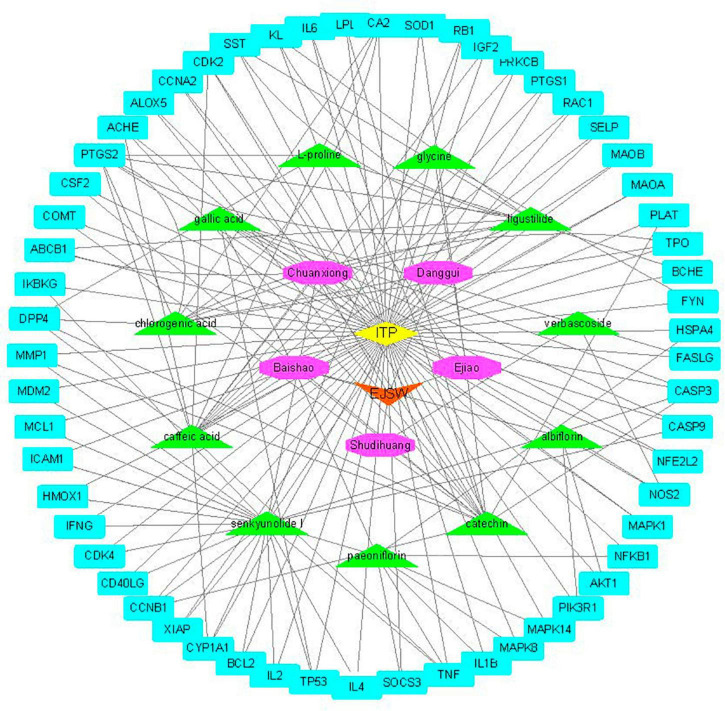
Disease-compound-target network of EJSW against. The blue rectangles represent targets. The green triangles represent the compounds. The purple octagons represents the five herbs of EJSW. The orange triangle represents EJSW and the yellow diamond represents ITP.

Active compounds of EJSW were identified by HPLC-DAD and LC-MS and their targets were obtained using HERB (see text footnote 1) and SwissTargetPrediction (see text footnote 2). Eleven of the 14 active compounds had targets, and the remaining 3e had no corresponding targets. Overlapping results from Gene Cards and CTD databases yielded 1726 ITP-related targets (Figure 3), and 58 common targets of ITP and EJSW were identified. A disease-compound-target network for EJSW treatment of ITP was constructed from the screened compounds and their targets (Figure 4). The network contained 76 nodes and 161 edges representing compound-target interactions.

### Protein-Protein Interaction Networks Analysis

**FIGURE 5:**
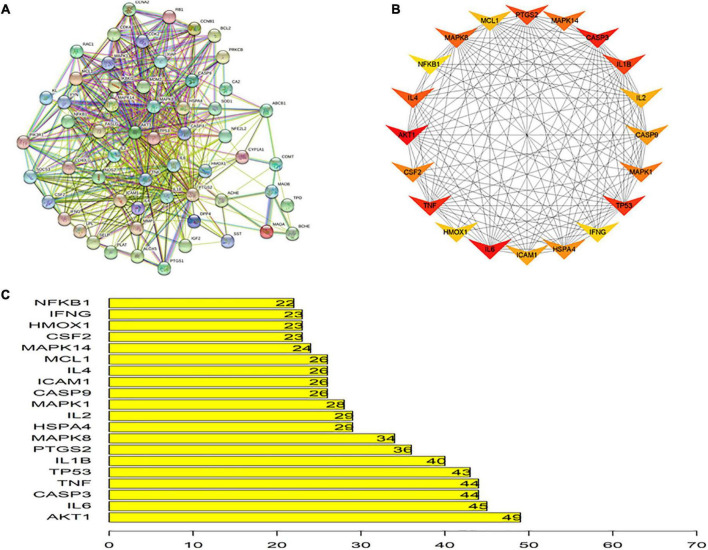
Protein-protein interaction network. **(A)** Protein-protein interaction network of putative targets of EJSW against ITP. **(B)** Top 20 of related targets. **(C)** The rank of top 20 related targets.

Protein-protein interactions are crucial for many biological processes including cell-to-cell interactions, metabolic control and developmental regulation, and studying PPIs is a primary objective of system biology (30). Therefore, a PPI network of putative ITP-related EJSW targets obtained from *in silico* analyses was generated, which included 58 nodes and 518 edges (Figure 5A). As shown in Figures 5B,C, the top 20 targets were represented as nodes and edges, and some targets including AKT1, TNF, IL-6, CASP3, and TP53 were strongly linked to EJSW treatment of ITP.

### Biological Process Analysis

**FIGURE 6:**
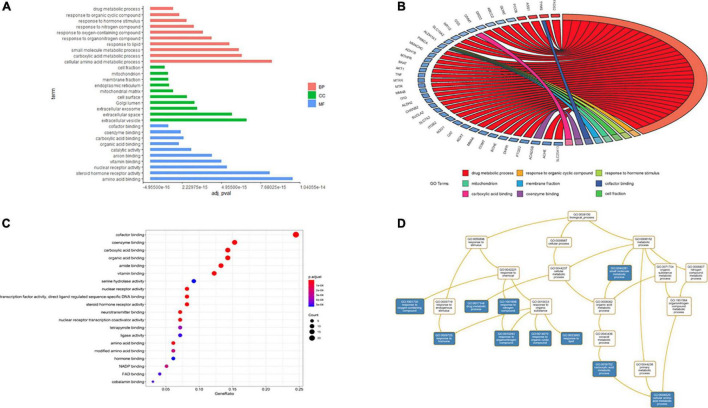
Gene ontology terms of candidate targets of EJSW against ITP. **(A)** The results of enrichment analysis containing BP, CC, MF. **(B)** The relationship of genes and GO terms (top3 of BP, CC and MF) based on the results of GO analysis. **(C)** The top 20 GO functional categories with *p* < 0.05 were selected. **(D)** The hierarchical relationships of GO terms enrichment.

**FIGURE 7:**
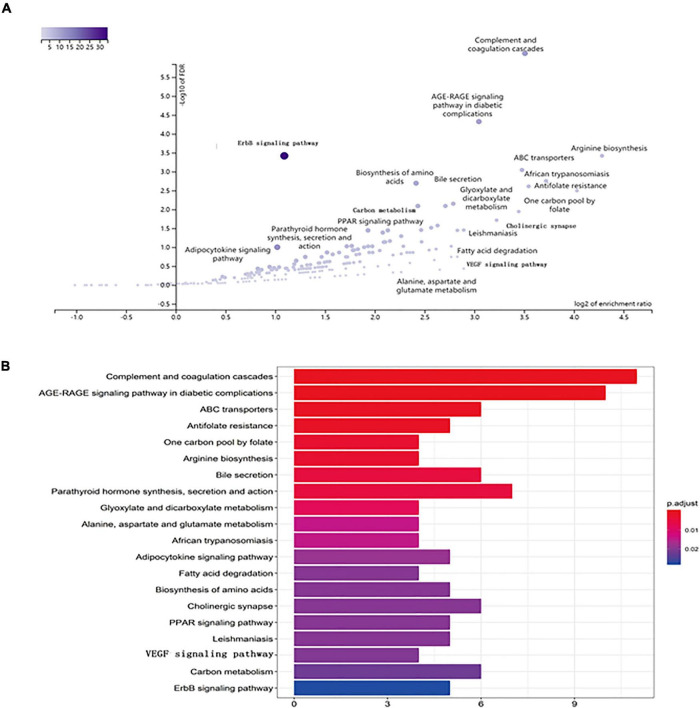
KEGG pathway enrichment of candidate targets of EJSW against ITP. **(A)** The volcano plot of KEGG pathway enrichment. **(B)** The top 20 pathways with *p* < 0.05 were selected.

The results of GO and KEGG pathway enrichment analyses (top 20, *p* < 0.05) are shown in Figures 6, 7. Highly enriched GO terms included cofactor binding, coenzyme binding, carboxylic acid binding, organic acid binding, amide binding, vitamin binding, and serine hydrolase activity. Analysis of hierarchical relationships for GO terms showed that small-molecule metabolic process was upstream and cellular amino acid metabolic process was downstream (Figure 6C).

**FIGURE 8:**
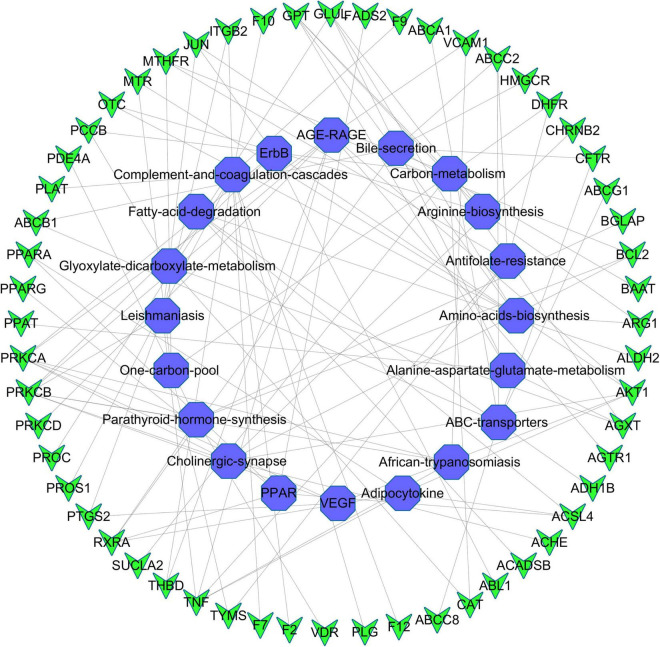
The top 20 pathways and the related gene targets by KEGG analysis. Purple symbols represent pathways and green were gene targets.

**FIGURE 9:**
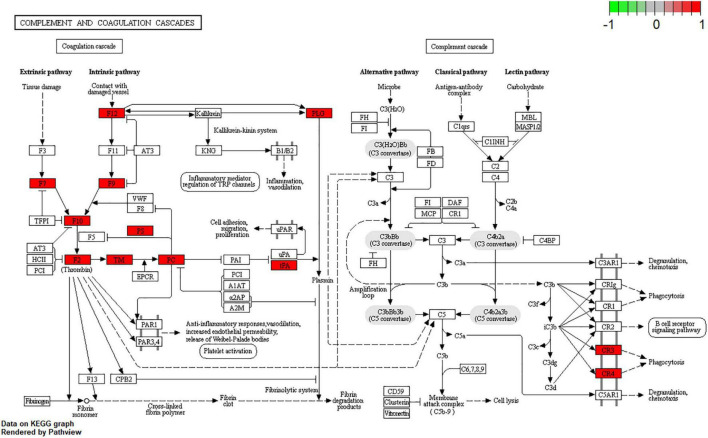
Complement and coagulation cascades signalling pathway. The red square represents targets of EJSW against ITP.

Kyoto Encyclopedia of Genes and Genomes pathway analysis was performed to identify pathways via which EJSW influenced ITP treatment. Twenty significantly enriched pathways were identified (*p* < 0.05), including complement and coagulation cascades, AGE-RAGE signalling in diabetic complications, ABC transporters, antifolate resistance, one-carbon pool related to folate, arginine biosynthesis, bile secretion, parathyroid hormone synthesis, secretion and action, glyoxylate and dicarboxylate metabolism, and VEGF signalling. In Figure 7 the length of the bar represents the number of genes and the colour represents the *p*-value. The distribution of targets in the 20 pathways through which EJSW affects ITP treatment are shown in Figure 8. The main targets of EJSW against ITP are associated with the complement and coagulation cascade signalling pathway (Figures 8, 9). In Figure 9, red squares represent targets of EJSW against ITP.

### Molecular Docking

**FIGURE 10:**
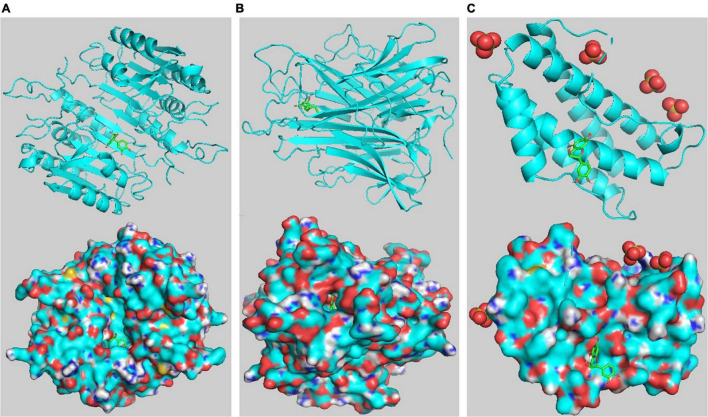
Target-compound interactions with the three highest molecular docking affinities. **(A)** CASP3- catechin. **(B)** TNF-senkyunolide I. **(C)** IL6- catechin. On the top shows the 3D structure of ligands and receptors, at the bottom shows the surface of the receptor and 3D structure of the ligands.

Five core targets (AKT1, TNF, IL-6, CASP3, and TP53) identified in the PPI network were validated by molecular docking with key compounds in EJSW. The results showed that the binding affinities of six key compounds to the core targets were below −5 kcal/mol (Table 2), which suggests that these six key components bind strongly to their core targets of ITP with high pharmacodynamic activity. These strong affinities suggest that EJSW may play a role in the treatment of ITP by acting on these core targets. The docking results for key compounds and core targets are shown in Figure 10.

**TABLE 2:** Molecular docking of active compounds and core targets in EJSW.

Target	Compounds	Affinity (kcal/mol)
AKT1	Albiflorin	−6.4
IL6	Catechin	−7.1
CASP3	Gallic acid	−5.5
CASP3	Senkyunolide I	−6.3
CASP3	Catechin	−7.7
TNF	Paeoniflorin	−6.8
TNF	Senkyunolide I	−7.2
TNF	Caffeic acid	−6.5
TP53	Gallic acid	−5.4
TP53	Senkyunolide I	−5.8

### Detection of Cytokines, Platelet Counts and Observation of Bleeding

**FIGURE 11:**
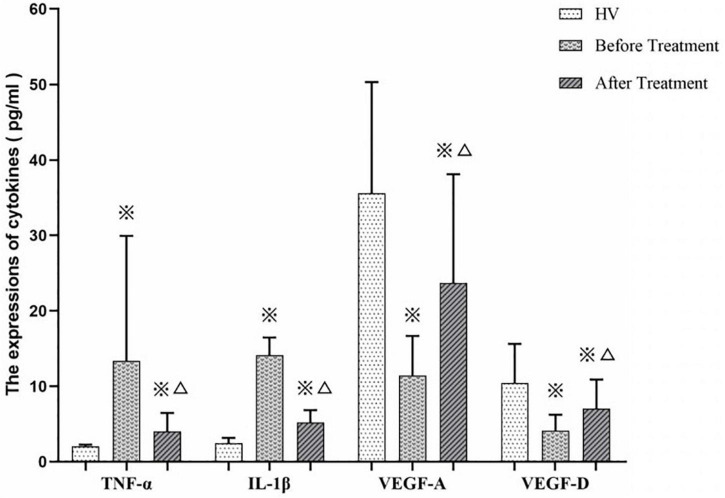
The expressions of TNF-α, IL-β, VEGF-A, and VEGF-D in ITP patients and HV.

We measured TNF-α, IL-1β, VEDF-A, and VEGF-D expression levels in ITP patients (before and after treatment by EJSW) and HV group members. As shown in Figure 11, expression of TNF-α (7.87 ± 5.59 vs. 2.04 ± 0.23) and IL-1β (14.11 ± 2.35 vs. 2.50 ± 0.67) was increased in ITP patients, while expression of VEGF-A (11.44 ± 5.23 vs. 35.59 ± 14.73) and VEGF-D (4.15 ± 2.10 vs. 10.46 ± 5.16) was decreased compared with HV. By contrast, after treatment with EJSW for 2 months, expression of TNF-α (4.05 ± 2.42 vs. 7.87 ± 5.59) and IL-1β (5.20 ± 1.65 vs. 14.11 ± 2.35) was decreased, while expression of VEGF-A (23.70 ± 14.40 vs. 11.44 ± 5.23) and VEGF-D (7.06 ± 3.85 vs. 4.15 ± 2.10) was increased. However, expression of TNF-α (4.05 ± 2.42 vs. 2.04 ± 0.23) and IL-1β (5.20 ± 1.65 vs. 2.50 ± 0.67) was still higher and expression of VEGF-A (23.70 ± 14.40 vs. 35.59 ± 14.73) and VEGF-D (7.06 ± 3.85 vs. 10.46 ± 5.16) was still lower than in the HV group.

**FIGURE 12:**
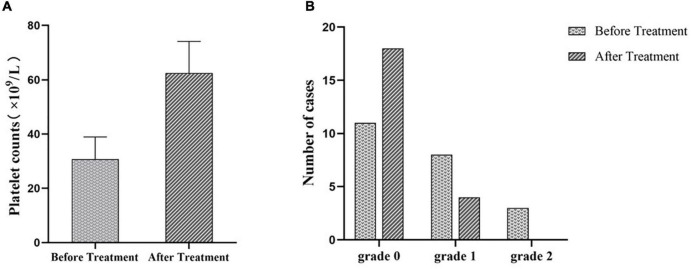
PLT counts and bleeding in patients with ITP after treatment with EJSW. **(A)** The platelet counts of ITP patients before and after treatment. **(B)** The number of cases of ITP patients with bleeding. Grade 0 = no bleeding; Grade 1 = petechiae; Grade 2 = mild blood loss.

Meanwhile, we measured the platelet counts of these 22 ITP patients before and after EJSW treatment. As shown in Figure 12A, platelet counts were significantly higher in patients with ITP after treatment with EJSW compared to before treatment (60 ± 11.63 vs. 31 ± 8.22, *p* < 0.05). We also recorded bleeding in ITP patients before and after treatment with EJSW. WHO Bleeding Scale were used to document and evaluate bleeding in patients. As shown in Figure 12B, the number of patients with bleeding was significantly reduced and bleeding was significantly improved with EJSW compared to before treatment [grade 0: 81.8% (18/22) vs. 50% (11/22); grade 1: 18.2% (4/22) vs. 36.4%(8/22); grade 2: 0%(0/22) vs. 13.6%(3/22), *χ^2^* = 6.023, *p* < 0.05].

## Discussion

Immune thrombocytopenia is an autoimmune hemorrhagic disease. Specifically, the destruction of platelets by the monocyte-macrophage system in ITP patients, resulting from the production of anti-platelet auto-antibodies, is relevant to immune disorders. However, a decrease in platelet count increases the risk of bleeding, and ITP patients present with petechiae in the skin and other bleeding symptoms, such as epistaxis and gingival bleeding. Purpura and ecchymosis can occur in any part of the skin or mucosa, and commonly in the lower extremities and distal upper extremities. Although glucocorticoids have many undesirable side effects, such as osteoporosis, femoral head necrosis, and psychiatric symptoms, they remain the first-line treatment for ITP (31, 32). However, TCM offers a holistic approach for ITP patient treatment and care. TCM believes that disease is the result of internal imbalance between the body’s energy (Yang) and a given substance (Yin) (15), and it rebalances energy and metabolic functions. Therefore, multi-ingredient TCM formulae are particularly suited for the treatment of complex diseases (33). The idea of network pharmacology is basically consistent with the holistic view of TCM, which helps to systematically and comprehensively explain the mechanism by which EJSW treats ITP.

The medical theory system of TCM has been used for thousands of years to treat diseases in China. Combining two or more compatible TCMs can typically improve the therapeutic effect in clinical treatment through synergism. According to TCM theory, ITP is thought to be a disease characterised by bleeding and blood deficiency, hence haemostasis and blood enrichment are necessary to treat ITP. However, it is common knowledge that platelets influence haemostasis. Thus, promoting platelet production is as important as preventing platelet destruction in view of modern medicine.

We identified 14 compounds in EJSW, of which gallic acid was able to intervenes in major inflammatory pathobiologies (34). Platelet-activating factor (PAF) is a potent inflammatory agonist while gallic acid partially protected mice from platelet-activating factor PAF-induced death (35). Caffeic acid inhibits IL-1β induced inflammation responses through suppression of NF-κB and MAPK related JNK signalling pathways (36). Verbascoside could also inhibit oxidative stress and inflammation (37, 38).

A network pharmacology approach can be used to probe the multi-component and multi-target characteristics of TCM compounds. In the present study, this technique was used to explore the mechanisms of EJSW in the treatment of ITP. The results showed a complex and interlaced network among target proteins of EJSW. Fifty-eight common targets of EJSW related to ITP were identified, 20 of which were major targets, including AKT1, IL6, CASP3, TNF, TP53, IL1B, PTGS2, MAPK8, HSPA4, IL2, MAPK1, CASP9, ICAM1, IL4, MCL1, MAPK14, CSF2, HMOX1, IFNG, and NFKB1, according to cutoff scores.

AKT1 can influence platelet activation (39), while TNF is involved in the regulation of a wide spectrum of biological processes including cell proliferation, differentiation, apoptosis, lipid metabolism and coagulation, and it has been implicated in autoimmune diseases (40). IL6 is a very important factor in determining Th17/Treg balance, which acts as a potent pro-inflammatory cytokine in T cells through promotion of Th17 differentiation and inhibiting Treg differentiation, and the control of IL-6 normalises the balance between Th17 and Treg and may alleviate autoimmune symptoms (41). CASP3, a major regulator of apoptosis, is a well-known execution protease, responsible for the final steps in the apoptotic pathway (42). And studies have demonstrated the presence of elevated apoptosis markers-activated caspase-3 in platelets and megakaryocytes from ITP patients, which could be reversed by treatment with intravenous immunoglobulin and prednisone (43, 44). TP53 is also associated with inflammation. Chronic inflammation in the presence of TP53 is inefficient at inducing carcinogenesis, on the contrary, chronic inflammation in the absence of TP53 generates acinar, ductal, neuroendocrine and sarcomatoid tumours (45).

Molecular docking validation indicated strong binding between key compounds and their targets. These targets are involved in multiple functions in diverse biological processes, and the functions of the main targets can be summarised as inflammatory responses, angiogenesis, vascular integrity, and platelet apoptosis.

We performed GO analysis of the targets for EJSW against ITP. The results of enrichment and target attribution analyses showed that EJSW regulates complex biological processes, including metabolic processes, biological regulation, and multicellular organismal processes. Enriched cellular components included membrane, membrane-enclosed lumen and vesicle subcategories. The main molecular functions included protein binding, ion binding, nucleotide binding and hydrolase activity.

Thus, EJSW regulates some key steps in metabolic and immune function processes, such as cofactor binding, carboxylic acid binding, transcription factor activity, direct ligand regulation, nuclear receptor activity, coenzyme binding, vitamin binding, organic acid binding, and others. These processes may be linked to immune disorders, vascular endothelium defects, and platelet apoptotic processes.

Immune thrombocytopenia is a type of autoimmune disease. Some genetic diseases can also lead to abnormal immune regulation and developed autoimmune haematological disorders, such as haemolytic anaemia and immune thrombocytopenia. But the genetic symptoms often have typical features and a clear genetic basis (46), which is not completely consistent with ITP we discussed. The present results showed that abnormal T-cell immunity leading to platelet destruction and platelet dyspoiesis is one of the main pathogenic mechanisms of ITP. Expression of Th1/Th2 as well as Th17/Treg are obviously imbalanced in ITP patients, and Th1 and Th17 are upregulated while Th2 and Treg are downregulated compared with healthy people (8, 47, 48). In addition, Th1 and Th17 are believed to induce inflammation while Th2 and Treg inhibit inflammation. When ITP occurs, the body is in an inflammatory state (49). Research shows that disorder of the apoptotic process also results in the pathogenesis of ITP (50). Unbalanced T-cells can also lead to disorder of apoptotic processes. The pathogenesis of ITP has been linked to gene expression, regulation of apoptosis, regulation of cell proliferation, nucleoplasm, transcription factor binding, histone deacetylase binding, protein kinase binding, and core promoter binding (51, 52), all of which were significantly enriched in the present study. Therefore, EJSW may treat ITP by regulating the pathogenesis of ITP.

The results of KEGG pathway analysis also showed that EJSW influences ITP through multiple pathways, including complement and coagulation cascades, AGE-RAGE signalling in diabetic complications, ABC transporters, antifolate resistance, ErbB signalling, and VEGF signalling. These pathways are closely related to cancer, metabolism, angiogenesis, inflammation, autoimmune diseases, coagulation, and other processes.

Complement and coagulation cascades are intimately associated with inflammatory responses and ensuring haemostatic maintenance (53). Indeed, the complement system plays a key role in components related to innate immunity. It shares numerous interactions with components of the haemostatic pathway, and organisms contain wounds and limit bleeding via by employing innate defence mechanisms (54, 55). It has been found that classical complement pathway can be activated by platelet autoantibodies and lead to the development of ITP (56–58), and complement activation is strongly associated with the severity of ITP (59). Therefore, complement pathway have become a new target for the treatment of ITP, and clinical trials have proven that classical complement pathway inhibitors can improve platelet counts in patients with chronic ITP with rapid and durable responses (60, 61). These fully illustrate that the complement pathway plays an important role in the pathogenesis of ITP.

The AGE- RAGE signalling pathway has strong links with vascular injury and inflammatory reactions (62, 63). Inflammatory factors in this signalling pathway, such as TNF and IL-1B, are related to vascular injury. In fact, these inflammatory factors can be considered inducers of platelet activation and immune imbalance in ITP (49, 64, 65). Retrospective correlation with clinical data suggests that platelet clearance is associated with inflammatory mediators (66, 67). Platelets are believed to play a central role in repairing vascular damage and stopping acute blood loss by tethering and adhering to sites of injury, recruiting other platelets and blood cells to the developing clot, releasing vasoactive small molecules and proteins, and assembling and activating plasma coagulation proteins in a tightly regulated temporal and spatial manner (68). In other words, platelets are essential for the maintenance of vascular endothelial integrity. Research has also shown that platelets are important mediators of inflammation, and they serve as important inducers of vascular cells via the complement system (69, 70).

The VEGF signalling pathway is mainly associated with angiogenesis. Vascular endothelial growth factors (VEGFs) constitute a sub-family of growth factors that stimulate the growth of new blood vessels (71). VEGFs are important signalling proteins involved in both vasculogenesis (the *de novo* formation of the embryonic circulatory system) and angiogenesis (the growth of blood vessels from pre-existing vasculature) (72). VEGF-A was the first member of the VEGF family to be identified, and the family also includes VEGF-B, VEGF-C, VEGF-D, and placenta growth factor (PlGF) (73, 74). VEGFs can reflect the state of blood vessel networks, and are closely linked to bleeding issues.

Thus, inflammatory factors, VEGFs, and immune imbalance are all correlated with the development of ITP. According to TCM theory, another function of EJSW is tonifying blood and arresting bleeding. The functions of the above target genes and signalling pathways conform to TCM theory. Therefore, the opinion that downregulating inflammatory factors is helpful to reduce the risk of bleeding is supported, due to restoration of immune balance and decreased platelet apoptosis. EJSW may treat ITP by inhibiting the expression of TNF-α, IL-1β, and other inflammatory factors, and by protecting vascular integrity through AGE-RAGE signalling, VEGF signalling, and complement and coagulation cascade signalling pathways. In the present study, TNF-α and IL-1β were upregulated in ITP, while VEGF-A and VEGF-D were downregulated. Conversely, after effective treatment with EJSW for 2 months, TNF-α and IL-1β were downregulated while VEGF-A and VEGF-D were upregulated. However, expression of TNF-α and IL-1β was still increased and expression of VEGF-A and VEGF-D was still decreased compared with normal controls. These results are consistent with those of earlier studies (75) reporting increased expressions of inflammatory factors including TNF-α and IL-1β and decreased expression of VEGF-A and VEGF-D in ITP patients. The present study also demonstrated that after treatment by EJSW, inflammation and vascular endothelial injury are relieved. Therefore, downregulating inflammatory factors can help to reduce the risk of bleeding. EJSW may treat ITP by inhibiting the expression of TNF-α, IL-1β, and other inflammatory factors, and by protecting vascular integrity through VEGF signalling, AGE-RAGE signalling, and complement and coagulation cascade signalling pathways.

However, this study has certain limitations. We only analysed data from a bioinformatic approach, and further specific *in vitro* and *in vivo* functional experiments are needed to validate the data. Overall, we explored the mechanisms and molecular targets of EJSW for treating ITP using network pharmacology and molecular docking. And our study found that the therapeutic activity of EJSW against ITP might be related to downregulating inflammatory factors and reducing vascular endothelial injury and regulating relevant pathways (VEGF signalling, AGE- RAGE signalling, and complement and coagulation cascade signalling pathways). Therefore, this study provide a scientific basis for clinical treatment of ITP, a direction for screening indicators of the follow-up clinical efficacy of EJSW, and new ideas for exploring the potential mechanisms of EJSW.

## Conclusion

The therapeutic activity of EJSW against ITP might be related to downregulating inflammatory factors and reducing vascular endothelial injury, which may enhance the platelet count. The effects of EJSW may involve regulating VEGF signalling, AGE- RAGE signalling, and complement and coagulation cascade signalling pathways. The results provide a scientific basis for clinical treatment of ITP, a direction for screening indicators of the follow-up clinical efficacy of EJSW, and new ideas for exploring the potential mechanisms of EJSW. We identified some core factors, but further screening and *in vivo* and *in vitro* experiments are needed to unequivocally identify the main regulatory targets of EJSW.

## Data Availability Statement

The data presented in this study are deposited in the Figshare repository: https://doi.org/10.6084/m9.figshare.20069915.v1.

## Ethics Statement

The studies involving human participants were approved by the Clinical Research Ethics Committee of Xiyuan Hospital, China Academy of Chinese Medical Sciences (Ethics approval number: 2015XLA108) and was conducted in compliance with the Declaration of Helsinki.

## Author Contributions

MW and XH: conceptualisation and design of the work, writing, review, and editing. MS, JM, ZW, XD, HC, XZ, and YSo: data acquisition and analysis. MW, YSu, and YSo: writing original draft preparation. All authors have read and agreed to the published version of the manuscript.

## Conflict of Interest

The authors declare that the research was conducted in the absence of any commercial or financial relationships that could be construed as a potential conflict of interest.

## Publisher’s Note

All claims expressed in this article are solely those of the authors and do not necessarily represent those of their affiliated organizations, or those of the publisher, the editors and the reviewers. Any product that may be evaluated in this article, or claim that may be made by its manufacturer, is not guaranteed or endorsed by the publisher.

## Footnotes

1
http://herb.ac.cn/

2
http://www.swisstargetprediction.ch/

3
https://www.genecards.org/

4
http://systemsdock.unit.oist.jp/iddp/home/index

5
https://cytoscape.org/

6
http://string-db.org/cgi/input.pl

7
https://www.R-project.org

8
http://www.webgestalt.org

9
http://www.bioconductor.org/

10
https://pubchem.ncbi.nlm.nih.gov/

11
https://www.rcsb.org/

12
https://pymol.org

13
https://autodock.scripps.edu/

14
https://vina.scripps.edu/
